# Effect of *PITX2* knockdown on transcriptome of primary human trabecular meshwork cell cultures

**Published:** 2011-05-05

**Authors:** Seyed Hassan Paylakhi, Jian-Bing Fan, Mohadeseh Mehrabian, Majid Sadeghizadeh, Shahin Yazdani, Ali Katanforoush, Mozhgan Rezaei Kanavi, Mostafa Ronaghi, Elahe Elahi

**Affiliations:** 1 Department of Genetics, Faculty of Biological Sciences, Tarbiat Modares University, Tehran, Iran; 2 Illumina Inc, San Diego, CA; 3 Department of Biotechnology, University of Tehran, Tehran, Iran; 4 Ophthalmic Research Center, Shahid Beheshti University of Medical Sciences, Tehran, Iran; 5 Department of Computer Science, Faculty of Mathematics, Shahid Beheshti University G.C., Tehran, Iran; 6 Central Eye Bank of Iran, Tehran, Iran; 7 Department of Biology, University College of Science, University of Tehran, Tehran, Iran; 8 Center of Excellence in Biomathematics, School of Mathematics, Statistics and Computer Science, College of Science, University of Tehran, Tehran, Iran

## Abstract

**Purpose:**

To identify genes whose expressions in primary human trabecular meshwork (TM) cell cultures are affected by the transcription factor pituitary homeobox 2 (PITX2) and to identify genes that may have roles in glaucoma. Known glaucoma causing genes account for disease in a small fraction of patients, and we aimed at identification of other genes that may have subtle and accumulative effects not easily identifiable by a genetic approach.

**Methods:**

Expression profiles derived using microarrays were compared between TM control cells and cells treated with *PITX2* siRNAs using three protocols so as to minimize false positive and negative results. The first protocol was based on the commonly used B statistic.  The second and third protocols were based on fold change in expression. The second protocol used a threshold of at least 2 fold change in expression, whereas the third protocol used ranking in fold change without setting a threshold. The likelihood of a selected gene being a true positive was considered to correlate with the number of protocols by which it was selected. By considering all genes that were selected by at least one protocol, the likelihood of false negatives was expected to decrease. Effects on a subset of selected genes were verified by real time PCR, western blots, and immunocytochemistry. Effects on *ALDH1A1*, were further pursued because its protein product, aldehyde dehydrogenase 1 family, member A1, has roles in oxidative stress and because oxidative stress is known to be relevant to the etiology of glaucoma.

**Results:**

The expression level of 41 genes was assessed by to be possibly affected by *PITX2* knockdown. Twenty one genes were down-regulated and twenty were upregulated. The expression of five genes was assessed to be altered by all three analysis protocols.  The five genes were *DIRAS3* (DIRAS family, GTP-binding RAS-like 3), *CXCL6* (chemokine (C-X-C motif) ligand 6), *SAMD5* (sterile alpha motif domain containing 5), *CBFB* (core-binding factor, beta subunit), and *MEIS2* (meis homeobox 2). Real time PCR experiments verified results on a subset of genes tested. Notably, the results were also confirmed in two independent TMs. Effects on *CXCL6* and *ALDH1A1* were also confirmed by western blots, and effects on *ALDH1A1* were further shown by immunocytochemistry. Data consistent with *PITX2* involvement in *ALDH1A1* mediated response to oxidative stress were presented.

**Conclusions:**

Bioinformatics tools revealed that the genes identified affect functions and pathways relevant to glaucoma. Involvement of *PITX2* in expression of some of the genes and in some of the pathways is being reported here for the first time. As many of the genes identified have not been studied vis-à-vis glaucoma, we feel they introduce new candidates for understanding this devastating disease.

## Introduction

Pituitary homeobox 2 (PITX2) is a homeobox transcription factor (TF) related to the paired class of homeodomain proteins [1]. It affects the development of various ocular tissues. During murine embryonic development, *PITX2* is expressed in neural crest and mesoderm precursors, both of which contribute to the periocular mesenchyme [2]. Mice carrying targeted deletions in *PITX2* exhibit eye development defects that include agenesis of the corneal epithelium and stroma, loss of extraocular muscles, and abnormalities of the optic nerve [3-5]. Finally, mutations in *PITX2* are cause of Axenfeld-Rieger syndrome (ARS) in a subset of patients [6,7]. ARS is characterized by defects in the anterior segment of the eye and systemic malformations [6,7]. Notably, approximately 50% of ARS patients develop glaucoma, usually in adolescence or early adulthood [8]. ARS patients harboring *PITX2* mutations are among those at risk of developing glaucoma [8].

The fact that mutations in *PITX2* can cause the ARS phenotype and that the mutations may culminate in a non-congenital form of glaucoma may be signatures of PITX2 functions in the mature TM [9]. Here, we report the effects of siRNA knockdown of *PITX2* on global gene expression in primary human TM cell cultures using high density microarrays. Pathways and functions implicated for the affected genes were derived using bioinformatics tools. *CXCL6* (chemokine (C-X-C motif) ligand 6) which has roles in immune response and *ALDH1A1* (aldehyde dehydrogenase 1 family, member A1) which has roles in oxidative stress were among the affected genes. The effects of the knockdown on *ALDH1A1* were further pursued [10,11].

## Methods

This research was performed in accordance with the Helsinki Declaration and with approval of the ethics board of the University of Tehran. Eye globes were obtained from the Central Eye Bank of Iran.

### Preparation of primary TM cultures

Four primary cultures were developed from donors without history of eye disease aged 25 (male; TM1), 30 (male; TM2), 65 (male; TM4), and 60 (female; TM5) years old at time of death [12]. Cells were maintained as previously described [12]. The nature of the cells was confirmed by demonstrating increased expression of myocilin mRNA and protein upon dexamethasone treatment (data not shown) [12].

### siRNA treatment and RNA extraction

Fourth to sixth passage cells were used in all experiments. Each of the four TM cultures was exposed to three siRNA treatments in duplicate wells. Lipofectamine RNAiMAX (Invitrogen, Carlsbad, CA) was used for reverse transfection according to the manufacturer’s instructions. We had previously shown that this treatment has no detectable effect on cell growth (data not shown). Approximately 8×10^4^ cells were added to wells containing siRNA to achieve a final siRNA concentration of 75 nM. These siRNA duplices (Dharmacon Research, Lafayette, CO) were used: *PITX2* siRNA-1 (J-017315–05), *PITX2* siRNA-2 (J-017315–06), and scrambled siRNA-1 (D-001810–01–20). All TM cultures had two independent treatments with scrambled siRNA (i.e., two treatments, two wells for each) so as to enable testing of technical replication of the array expression assessments.

Forty eight hours after exposure to siRNAs, cells were harvested and cells of duplicate wells were combined and placed in RNX^TM^-plus (Cinnagen, Tehran, Iran). Total RNA was isolated according to the manufacturer’s instructions. RNA quality was assessed using density ratio of 28S to 18S rRNA bands. Half of the RNA was set aside for microarray experiments and the other half was used for real time PCR.

### Assessment of knockdown by siRNAs

Candidate control genes for assessment of effects of *PITX2* siRNAs were *ACTB*  (actin, beta), *β2M*  (beta-2 microglobulin), *GAPDH*  (glyceraldehyde-3-phosphate dehydrogenase), and *HPRT1* (hypoxanthine phosphoribosyltransferase 1). cDNA synthesis was done by standard procedures and real time PCR was performed on a Corbett 65H0 machine (Corbett Research, Sidney, Australia) using the QuantiFast SYBR Green PCR Kit (QIAGEN, Germantown, MD). After selection of appropriate control gene using GNorm program [13], *PITX2* siRNA knockdown effects on *PITX2* were determined by real time PCR. The real time PCR primers were obtained from QIAGEN.

### Microarray experiments and data analysis

Array experiments were performed only on TM1 and TM2 cultures using HumanRef-8 V2 Illumina Genome-Wide Expression BeadChips according to the manufacturer’s instructions (Illumina, San Diego, CA). Arrays were scanned with the Illumina BeadArray reader and images were analyzed using Beadstudio VI software. The Beadstudio data were normalized using a quantile based algorithm available in the Beadarray software [14]. Values for all genes were compared between the two replicate control samples of TM1 and between the two replicate control samples of TM2. Similarly, effect of *PITX2* siRNA treatments on three housekeeping genes *ACTB*, *β2M*, and *GAPDH* on the different arrays were determined.  Subsequently, genes the expressions of which were affected by siRNA treatments were assessed based on three protocols as described below.

First, the Limma package was used [15]. For each of the four assessments (two siRNAs, two TMs), genes were ordered based on the value of their associated B statistic. Genes were then selected on conditions that they had a B statistic value ≥2 in at least two of the four assessments, that both siRNAs were represented in these assessments, and that the direction of change in expression was the same in the assessments.

In the second protocol, expression in presence of each of two siRNAs was compared separately to each of two controls for each TM, producing eight comparisons for the two TMs. Genes were selected on conditions that they showed ≥2 fold change in the same direction in at least four of the eight comparisons and that each siRNA was represented at least once among the selected comparisons.

      In the third protocol, essentially the 40 top ranking genes affected by siRNAs of *PITX2* based on fold change in expression were selected. Initially, fold changes in the presence of siRNA1 in each TM were assessed by comparison to average of controls for the respective TM, and forty top ranking genes in each TM were identified (Group A genes: siRNA-1 top 40 TM1 list,  siRNA-1 top 40 TM2 list). Subsequently, genes affected were sorted by adding rank of genes affected in the two TMs, and selecting the top 40 genes (Group A genes: siRNA-1 top 40 TM1/2 list). This process was repeated for siRNA-2 (Group B genes: siRNA-2 top 40 TM1 list, siRNA-2 top 40 TM2 list, siRNA-2 top 40 TM1/2 list). Finally, the rankings of genes affected by both siRNAs in each TM were added, and then the rankings of both siRNAs in both TMs were added (Group C genes: siRNA-1/2 top 40 TM1 list, siRNA-1/2 top 40 TM2 list, siRNA-1/2 top 40 TM1/2 list).  In this protocol, genes were ultimately selected that appeared at least once in each of the three Groups of genes. A computer program written to perform this selection task is available at Joint-rank.

### Real time PCR

Real time PCR was performed on a subset of microarray based selected genes on the two TMs that had been used for microarray analysis and also on TM4 and TM5. cDNA synthesis and real time PCR were performed as described above using QIAGEN primers for *ACTB, ALDH1A1, CXCL6, DIRAS3* (DIRAS family, GTP-binding RAS-like 3), *DKK1* (Dickkopf-1), *KCNJ2* (potassium inwardly-rectifying channel, subfamily J, member 2), *MEIS2* (meis homeobox 2), *PITX2,* and *SAMD5* (sterile alpha motif domain containing 5). At least four replicate PCRs for each gene in each TM were performed. Detailed information on all primers is provided in Table 1. *ACTB* was used as control gene in the real time PCR experiments except where indicated otherwise. Fold change in expression of selected genes were obtained using software provided by the Corbett instrument, used for the real time PCR experiments..

**Table 1:** Primers used in real time PCR experiments.

Gene*	**Entrez gene ID**	**QIAGEN Cat. #**	**Target transcript**	**Amplified exons**	**Amplicon length (bp)**
*ACTB*	60	QT01680476	NM_001101	**	104
*ALDH1A1*	216	QT00013286	NM_000689	11, 12	97
*B2M*	567	QT00088935	NM_004048	1,2	98
*CXCL6*	6372	QT00211155	NM_002993	2	75
*DIRAS3*	9077	QT00040558	NM_004675	3,4	60
*DKK1*	22943	QT00009093	NM_012242	2,3	137
*GAPDH*	2597	QT01192646	NM_002046	1,2,3	119
*KCNJ2*	3759	QT00001022	NM_000891	1,2	150
*MEIS2*	4212	QT00077315	NM_002399	6,7,8	143
*PITX2*	5308	QT01006033	NM_153426	3,4	104
*SAMD5*	389432	QT01154223	NM_001030060	1,2	105

*HPRT primers were designed by us. **Data not provided by QIAGEN.

### Western blot analysis

TM cultured cells were harvested and lysed in Lameli lysis buffer. Protein (60–100 µg) was electrophoresed on 10%–15% denaturing polyacrylamide gels and the resolved proteins were transferred onto nitrocellulose membranes. The ECL Advance Luminol based chemiluminescence detection kit was used for detection of specific proteins after reaction with appropriate antibodies according to the manufacturer’s instructions (GE Healthcare, Salt Lake City, UT). Specific antibodies used were goat polyclonal antibodies (Santa Cruz Biotechnology, Inc., Santa Cruz, CA) against human myocilin (C-15), Lamin B (C-20) and rabbit polyclonal antibodies (Abcam, Cambridge, MA)  against ALDH1A1 (ab51028) and CXCL6 (ab9923). Lamin served as control protein. Secondary antibodies were horseradish peroxidase conjugated anti-goat (SC-2768; Santa Cruz Biotechnology) and anti-rabbit (Sigma-Aldrich, Poole, UK) antibodies. Western blot analysis for each protein was performed twice.

### Immunofluorescence analysis on cultured cells and histological sections

TM1 cells grown on coverslips in the presence of scrambled siRNA or *PITX2* siRNA-1 were fixed with an acetone-methanol mix, blocked with BSA, incubated with the anti-ALDH1A1 antibody described above, and exposed to fluorescent conjugated goat anti-rabbit secondary antibody (AF8035; Razi BioTech, Tehran, Iran). Nuclei were counterstained with DAPI (Invitrogen). Cells were visualized with an Axioplan 2 fluorescence microscope (Carl Zeiss, Jena, Germany). Additionally, expression of both ALDH1A1 and CXCL6 were assessed in cryosections of a globe from a three year old cadaver.

### Comparison of genes identified to genes reported in previous glaucoma relevant array studies

Fifteen microarray gene expression studies in which effects of various glaucoma relevant conditions or treatments were studied, including effects of dexamethsone, pressure, TGFβ, and gene knockdown, have been reported [16-30]. *PITX2* affected genes identified here were compared to genes identified in the previous studies to identify possible commonalities.

### In silico analysis of selected genes

Promoter regions of selected genes were screened for PITX2 binding sites so as to identify genes whose transcriptions are more likely to be directly affected by this TF. PITX2 binds the bicoid sequence element 5ʹ-TAATCC-3ʹ [31,32]. The region between 3,000 bp upstream and 200 bp downstream of transcription initiation sites of potential target genes was screened for presence of the element [33,34]. Finally, genes identified by the microarray analysis were analyzed to identify potentially relevant functional pathways and functional categories or gene ontology terms annotated by Kyoto Encyclopedia of Genes and Genomes (KEGG) [35] and Gene Ontology (GO) [36] using the Database for Annotation, Visualization, and Integrated Discovery (DAVID) bioinformatics tool [37]. A cutoff p value of 0.05 was used for enriched KEGG pathways or GO functions.

### Hydrogen peroxide (H_2_O_2_) and lithium chloride (LiCl) treatments

H_2_O_2_ and LiCl treatments were used, respectively, as surrogates for analysis of effects of oxidative stress and Wnt signaling [38,39]. For assessment of these agents on expression of *PITX2* and *ALDH1A1*, TM1 cells were exposed to five treatments and each treatment had its own control. The first treatment was exposure to *PITX2* siRNA, and control treatment was exposure to scrambled siRNA. In the second, cells were exposed to 600 mM H_2_O_2_ for 4 h, and in the third treatment cells had the same exposure to H_2_O_2_ after having been exposed to PITX2 siRNA-1. The fourth treatment was exposure to 20 mM LiCl for 12 h. In the fifth treatment, H_2_O_2_ was added to cells that had already been exposed to LiCl for 12 h, and incubation was continued for an additional 4 h. After treatments, real time PCR was performed to assess levels of *PITX2* and *ALDH1A1* expression. *β2M* was used as control gene because H_2_O_2_ has been reported to affect actin expression [39]. The effects of the H_2_O_2_ and LiCl treatments on *ALDH1A1* expression were also assessed by western analysis. Statistical analysis was done using Relative Expression Software Tool (REST) software [40].

## Results

**Figure 1:**
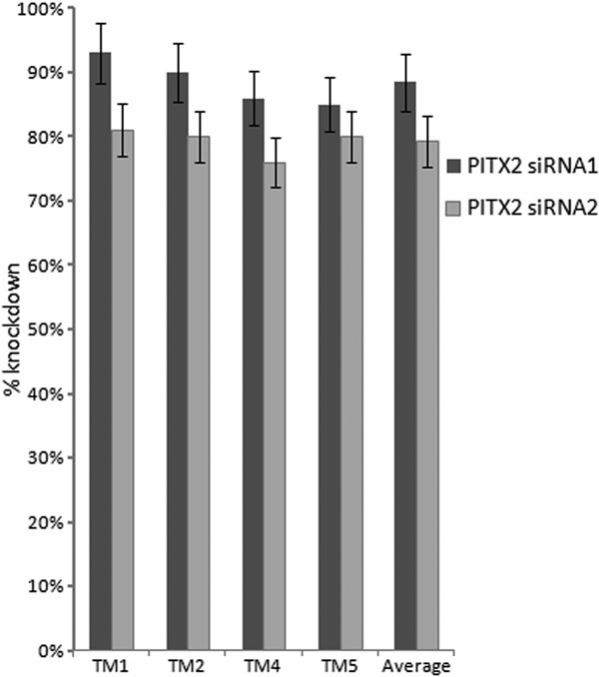
*PITX2* transcription knockdown by *PITX2* siRNA-1 and *PITX2* siRNA-2 as assessed by real time PCR.

GNorm identified *ACTB* and *β2M* as genes with best stability value (M=0.028).  The amount of knockdown of *PITX2* mRNA by its siRNAs using *ACTB* as control in the four TM cultures (TM1, TM2, TM4, TM5) ranged from 76%–93% and the average was 84% as assessed by real time PCR (Figure 1). The raw microarray data on TM1 and TM2 have been deposited in the Gene Expression Omnibus (GEO) database, (accession GSE27275). The levels expression of most genes in the two independent TM1 culture control samples that had been treated in identical manner with scrambled siRNA were very similar and only one gene showed >│2.0│fold change in expression between the two replicates (*GTF21*; 2.1×). Similarly, only one gene showed >│2.0│fold change in expression between the two independent scrambled siRNA treated TM2 cells (*TAF5L*; 2.1×). These results are indicative of acceptable experimental replication. Fold change in expression of three housekeeping genes *ACTB*, *β2M*, and *GAPDH*  due to *PITX2* siRNA treatments was low for the siRNAs in both TM1 and TM2, averaging 1.01 (Table 2).

**Table 2:** Effect of *PITX2* siRNAs on housekeeping genes *ACTB, β2M*, and *GAPDH* based on microarray data.

**Gene**	TM1*	TM2*	Average**
*ACTB*	0.85	0.86	0.85
*B2M*	1.13	1.23	1.18
*GAPDH*	0.98	0.98	0.98

*Average effect of 2 siRNAs on each TM. **Average effect of 2 siRNAs on TM1 and TM2.

### Microarray data analysis

Protocols 1, 2, and 3 identified, respectively, 26, 14, and 20 genes affected by *PITX2* siRNA treatments, and some genes were common between genes selected by different protocols. In total 41 different genes were identified, and these will henceforth be called “*PITX2*-filtered genes.” Nine genes were identified by two protocols and five by all three of the protocols.  The genes identified are listed in Table 3. The five genes identified by all the protocols were *DIRAS3*, *CXCL6*, *SAMD5*, *CBFB* (core-binding factor, beta subunit), and *MEIS2*. Known glaucoma causing genes, *CYP1B1* (cytochrome P450, family 1, subfamily B, polypeptide 1), *MYOC* (myocilin), *OPTN* (optineurin), and *WDR36* (WD repeat domain 36) were not among the genes affected by PITX2 [41]. *LTBP2* (latent transforming growth factor beta binding protein 2), a primary glaucoma causing gene, was not probed on the arrays [42].

**Table 3:** Microarray identified genes with changed expression due to *PITX2* siRNA treatments.

**Genes identified based on B statistic**					**Genes identified based on twofold change cut-off**			**Genes identified based on rank in fold change without cut-off**			**All genes selected by one or more protocol**					
**(Protocol 1)**					**(Protocol 2)**			**(Protocol 3)**			**(protocol 1, 2, +/or 3)**					
**Gene**	**B value**	**P value**	**mRNA fold change**		**Gene**	**mRNA fold change**		**Gene**	**mRNA fold change**		**Gene**	mRNA fold change*		**P 1**	**P 2**	**P 3**
** **	** **	** **	**Down**	**Up**	** **	**Down**	**Up**	** **	**Down**	**Up**	** **	**Down**	**Up**	** **	** **	** **
*DIRAS3*	5.9	0.013848	4.2	** **	*DIRAS3*	3.3	** **	*DIRAS3*	4.2	** **	*DIRAS3*	3.9	** **	X	X	X
*CXCL6*	4.1	0.019535	3.1	** **	*CXCL6*	2.9	** **	*CFL2*	** **	3.5	*CXCL6*	3.3	** **	X	X	X
*SAMD5*	4.4	0.014213	3	** **	*CFL2*	** **	2.8	*SAMD5*	3	** **	*CFL2*	** **	3.2	** **	X	X
*XYLT1*	4.4	0.082004	2.9	** **	*KHDRBS3*	2.7	** **	*CXCL6*	3	** **	*SAMD5*	2.9	** **	X	X	X
*KHDRBS3*	4.6	0.008829	2.8	** **	*SAMD5*	2.7	** **	*ALDH1A1*	2.5	** **	*XYLT1*	2.9	** **	X	** **	** **
*ALDH1A1*	3.6	0.030613	2.5	** **	*ADAMTS5*	** **	2.5	*CBFB*	2.3	** **	*KHDRBS3*	2.8	** **	X	X	** **
*C7orf47*	2.7	0.048148	2.3	** **	*C7orf47*	2.4	** **	*PLP2*	** **	2.2	*ALDH1A1*	2.5	** **	X	** **	X
*LOC653602*	4.2	0.040201	** **	2.3	*TMEM65*	** **	2.4	*TMEM65*	** **	2.1	*ADAMTS5*	** **	2.5	** **	X	** **
*CBFB*	2.1	0.054419	2.3	** **	*CBFB*	2.4	** **	*AUH*	** **	2	*PATZ1*	2.4	** **	** **	X	** **
*C7orf63*	4.2	0.070604	** **	2.2	*PATZ1*	2.4	** **	*MEIS2*	** **	2	*CBFB*	2.4	** **	X	X	X
*PLP2*	3.2	0.02711	** **	2.2	*LOC653602*	** **	2.3	*BHLHB3*	2	** **	*C7orf47*	2.3	** **	** **	X	X
*GRP*	3.6	0.036988	2.1	** **	*BBS5*	** **	2.2	*PIP4K2B*	** **	1.9	*LOC653602*	** **	2. 3	** **	X	X
*PMS2*	3.82	0.032	** **	2.1	*CTXN1*	** **	2.2	*FLG*	1.9	** **	*TMEM65*	** **	2.3	** **	X	X
*SMC2*	3.5	0.063532	2	** **	*MEIS2*	** **	2.2	*LIN7A*	1.8	** **	*PLP2*	** **	2.2	X	** **	X
*MEIS2*	3.2	0.068199	** **	2	** **	** **	** **	*HAPLN1*	1.7	** **	*MEIS2*	** **	2.1	X	X	X
*BHLHB3*	3.3	0.024836	2	** **	** **	** **	** **	*GALNT1*	1.7	** **	*CTXN1*	** **	2.2	** **	X	** **
*GBP2*	3	0.039074	** **	2	** **	** **	** **	*QDPR*	** **	1.6	*C7orf63*	** **	2.2	X	** **	** **
*IRS2*	3.4	0.005579	2	** **	** **	** **	** **	*PMS2*	** **	1.5	*BBS5*	** **	2.2	** **	X	** **
*LMNB1*	3.5	0.00633	2	** **	** **	** **	** **	*NOMO2*	** **	1.5	*GRP*	2.1	** **	X	** **	** **
*SLC12A2*	2.9	0.04161	1.9	** **	** **	** **	** **	*MICALL2*	** **	1.4	*GBP2*	** **	2	X	** **	** **
*CEBPG*	2.5	0.061234	** **	1.9	** **	** **	** **	** **	** **	** **	*BHLHB3*	2	** **	X	** **	X
*CD4*	2.9	0.039381	** **	1.8	** **	** **	** **	** **	** **	** **	*LMNB1*	2	** **	X	** **	** **
*ELK3*	2.6	0.012323	1.8	** **	** **	** **	** **	** **	** **	** **	*IRS2*	2	** **	X	** **	** **
*FAM70A*	2.9	0.039441	1.8	** **	** **	** **	** **	** **	** **	** **	*SMC2*	2	** **	X	** **	** **
*GBP5*	4.3	0.032596	** **	1.8	** **	** **	** **	** **	** **	** **	*AUH*	** **	2	** **	** **	X
*PTPRR*	2.4	0.013709	** **	1.8	** **	** **	** **	** **	** **	** **	*PIP4K2B*	** **	1.9	** **	** **	X
** **	** **	** **	** **	** **	** **	** **	** **	** **	** **	** **	*CEBPG*	** **	1.9	X	** **	** **
** **	** **	** **	** **	** **	** **	** **	** **	** **	** **	** **	*SLC12A2*	1.9	** **	X	** **	** **
** **	** **	** **	** **	** **	** **	** **	** **	** **	** **	** **	*FLG*	1.9	** **	** **	** **	X
** **	** **	** **	** **	** **	** **	** **	** **	** **	** **	** **	*CD4*	** **	1.8	X	** **	** **
** **	** **	** **	** **	** **	** **	** **	** **	** **	** **	** **	*ELK3*	1.8	** **	X	** **	** **
** **	** **	** **	** **	** **	** **	** **	** **	** **	** **	** **	*LIN7A*	1.8	** **	** **	** **	X
** **	** **	** **	** **	** **	** **	** **	** **	** **	** **	** **	*PTPRR*	** **	1.8	X	** **	** **
** **	** **	** **	** **	** **	** **	** **	** **	** **	** **	** **	*FAM70A*	1.8	** **	X	** **	** **
** **	** **	** **	** **	** **	** **	** **	** **	** **	** **	** **	*GBP5*	** **	1.8	X	** **	** **
** **	** **	** **	** **	** **	** **	** **	** **	** **	** **	** **	*PMS2*	** **	1.8	X	** **	X
** **	** **	** **	** **	** **	** **	** **	** **	** **	** **	** **	*GALNT1*	1.7	** **	** **	** **	X
** **	** **	** **	** **	** **	** **	** **	** **	** **	** **	** **	*HAPLN1*	1.7	** **	** **	** **	X
** **	** **	** **	** **	** **	** **	** **	** **	** **	** **	** **	*QDPR*	** **	1.6	** **	** **	X
** **	** **	** **	** **	** **	** **	** **	** **	** **	** **	** **	*NOMO2*	** **	1.5	** **	** **	X
** **	** **	** **	** **	** **	** **	** **	** **	** **	** **	** **	*MICALL2*	** **	1.4	** **	** **	X

*Average fold change of protocols.

**Figure 2:**
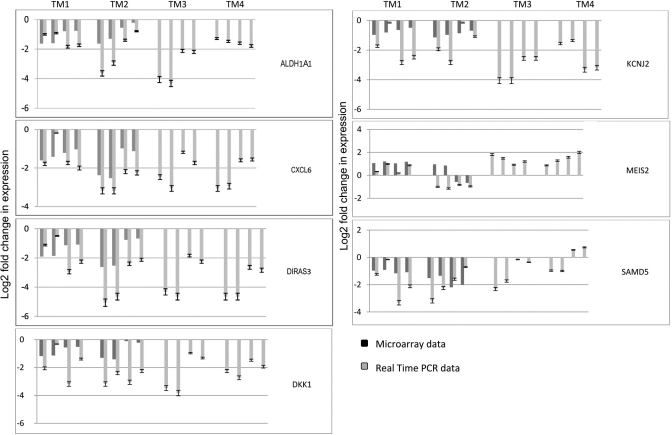
Real time PCR confirmation of selected genes affected by *PITX2* siRNAs. All genes tested were among *PITX2*-filtered genes except *DKK1* and *KCNJ2*.  For each TM, the four pair of bars show log_2_ fold change in expression based on data of each of two *PITX2* siRNAs compared to each of two controls exposed to scrambled siRNA.  Standard deviations for real time PCR data are shown.  See text for further details on real time PCR experiments.

To confirm microarray based assessments, real time PCR was performed on a subset of genes. Five genes affected by *PITX2* siRNAs, *ALDH1A1*, *CXCL6, DIRAS3, MEIS2, SAMD5,* and two additional candidate genes, *DKK1* and *KCNJ2*, were tested. The latter two were not among the genes identified by the array analysis protocols; they were selected for assessment by real time PCR based on earlier reports suggesting PITX2 may affect their expressions [17,43,44]. Effects of *PITX2* siRNAs as assessed by real time PCR corroborated the array data of most genes tested in the sense of the genes being down-regulated or upregulated (Figure 2). The only exception was *MEIS2* in TM2. Notably, the effects on the genes observed in TM1 and TM2, were also observed in TM4 and TM5 which originated from globes of older individuals. *DKK1* and *KCNJ2* that had not been selected by criteria of the array analysis protocols were also shown by real time PCR to be affected by *PITX2* knockdown. Although the array data on these two genes did not meet the protocols selection criteria, the array data did in fact indicate some down-regulation consistent with the real time PCR results (Figure 2).

**Figure 3:**
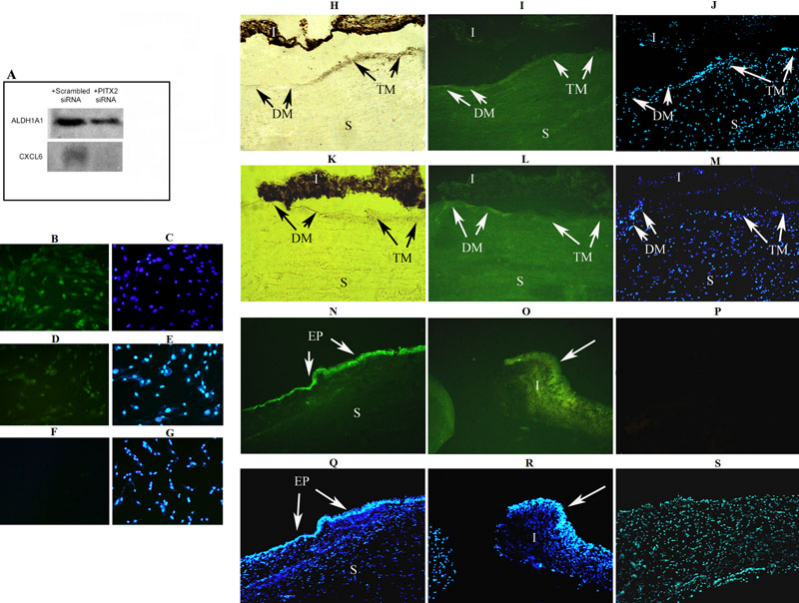
Confirmation of effects of *PITX2* knockdown on *ALDH1A1* and *CXCL6* at the protein level. Results shown are with PITX2 siRNA-1 and TM1. **A**: Representative western immunoblots of ALDH1A1 and CXCL6 in protein extracts of TM cultures exposed to scrambled siRNA and PITX2 siRNA. **B**-**G**: Immunofluorescent analysis of ALDH1A1expression in TM cultured cells exposed to scrambled siRNA (**B**, **C**) and *PITX2* siRNA (**D**, **E**). In cells treated with scrambled siRNA (**B**), ALDH1A1 expression is apparent in cytoplasm and nucleus of most cells. However, decreased expression is evident in many *PITX2* siRNA treated cells (**D**). No immunofluorescence is observed in the negative control (**F**). DAPI stained cells are also shown (**C**, **E**, **G**). **H**-**S**: Immunohistochemical demonstration of ALDH1A1 and CXCL6 expression in the human eye. Cryosections of anterior section of donated globe observed under light microscope (**H**, **K**), by immunofluoresence after staining for ALDH1A1 and CXCL6 (**I**, **L**, **N**, **O**), and after staining with DAPI (**J**, **M**, **Q**, **R**) are shown. **H**, **I**, **J**: sections stained with anti-ALDH1A1. **K**, **L**, **M**: sections stained with anti-CXCL6. Expression of both ALDH1A1 and CXCL6 in the trabecular meshwork (TM) and stroma (S), and higher expression in the Descemet membrane (DM) are evident. **N**, **Q**: highest expression of ALDH1A1 was observed in the epithelium of the cornea (EP). **O**, **R**: highest expression of CXCL6 was observed in the iris (I), particularly in the iris epithelium. **P**, **S**: negative control shows no immunofluorescent staining. Optical lens magnification 5× (all except **O** and **R**), 20× (**O** and **R**).

*CXCL6* and *ALDH1A1,* were further analyzed by western blotting, and the results evidenced that *PITX2* siRNAs affected decreased expression of CXCL6 and ALDH1A1 at the protein level (Figure 3A). Furthermore, decreased expression of ALDH1A1 protein in *PITX2* siRNA treated cells was also shown by immunofluorescence analysis of the treated cells (Figure 3B-G). Immunofluorescence on globe sections showed expression of both genes in the trabecular meshwork, and additionally in the stroma and Descemet membrane (Figure 3H-S). Highest expressions of CXCL6 and ALDH1A1 were observed, respectively in the iris epithelium and corneal epithelium.

Of the 41 *PITX2*-filtered genes identified by analysis of array data, 11 were also identified in one or more previously reported glaucoma related global gene expression studies (Table 4).  Notably, *DIRAS3* and *CXCL6* that had shown the two highest fold changes in our experiments (3.9X and 3.3×, respectively; Table 3) and that had both been selected on the basis of all three selection protocols, had also been repeatedly reported in other studies [17-20]. *ADAMTS5* that was identified by only one of our selection protocols was previously reported in three studies [17,21,22]. *DKK1* and *KCNJ2* which were shown to be affected by *PITX2* siRNAs by real time PCR experiments were each previously reported in one study [16,22].

**Table 4:** *PITX2* affected genes identified in other glaucoma related global gene expression studies*.

***PITX2* filtered genes**	**Number of other studies in which affected gene identified**	**Reference**
*CXCL6*	3	[18-20]
*ADAMTS5*	3	[17,21,22]
*DIRAS3*	2	[17,19]
*BBS5*	1	[17]
*ELK3*	1	[29]
*GRP*	1	[21]
*HAPLN1*	1	[18]
*MICALL2*	1	[17]
*PLP2*	1	[30]
*SLC12A2*	1	[18]
*ALDH1A1*	1	[27]
DKK1**	1	[22]
KCNJ2**	1	[16]

*PITX2 filtered genes reported here were sought in lists of selected genes of the array studies reported in references [16-30]. **These 2 included were based on real time PCR results.

### In silico analysis of selected genes

The PITX2 binding element was identified within regions surrounding the transcription initiation sites of some of the affected genes. Of the 41 *PITX2* filtered genes, 18(43.9%) contained two or more of the binding sequences (data not shown). For example, *DKK1* and *MEIS2* had, respectively, four and three binding sites. Table 5 lists KEGG and GO terms that are enriched among the 41 *PITX2* affected genes.

**Table 5:** Functional annotations enriched for genes affected by *PITX2*  siRNAs*.

**Category**	**GO ID and Term**	**Number of genes**	**p value**
GOTERM_CC_ALL	GO:0016363~nuclear matrix	2	0.0013123
GOTERM_BP_ALL	GO:0030098~lymphocyte differentiation	4	0.0013564
GOTERM_CC_ALL	GO:0034399~nuclear periphery	2	0.001421
GOTERM_CC_ALL	GO:0005789~endoplasmic reticulum membrane	3	0.0014526
KEGG_PATHWAY	hsa04666:Fc gamma R-mediated phagocytosis	2	0.0020273
GOTERM_BP_ALL	GO:0002520~immune system development	5	0.0026937
GOTERM_BP_ALL	GO:0002521~leukocyte differentiation	4	0.0026975
GOTERM_MF_ALL	GO:0019901~protein kinas binding	3	0.005062
GOTERM_MF_ALL	GO:0030246~carbohydrate binding	4	0.0055687
GOTERM_MF_ALL	GO:0019900~kinase binding	3	0.0071612
GOTERM_BP_ALL	GO:0046649~lymphocyte activation	4	0.0086461
GOTERM_BP_ALL	GO:0030097~hemopoiesis	4	0.0137107
GOTERM_BP_ALL	GO:0006955~immune response	6	0.0145876
GOTERM_BP_ALL	GO:0045321~leukocyte activation	4	0.0146618
GOTERM_CC_ALL	GO:0042175~nuclear envelope-endoplasmic reticulum network	3	0.0158339
GOTERM_CC_ALL	GO:0070160~occluding junction	2	0.0167652
GOTERM_CC_ALL	GO:0005923~tight junction	2	0.0167652
GOTERM_BP_ALL	GO:0048534~hemopoietic or lymphoid organ development	4	0.0177369
GOTERM_CC_ALL	GO:0043232~intracellularnon-membrane-bounded organelle	10	0.0186364
GOTERM_CC_ALL	GO:0043228~non-membrane-bounded organelle	10	0.0186364
GOTERM_CC_ALL	GO:0009898~internal side of plasma membrane	3	0.0186943
GOTERM_CC_ALL	GO:0044432~endoplasmic reticulum part	3	0.0215332
GOTERM_CC_ALL	GO:0005783~endoplasmic reticulum	5	0.0216708
GOTERM_CC_ALL	GO:0043296~apical junction complex	2	0.0220511
GOTERM_CC_ALL	GO:0016327~apico lateral plasma membrane	2	0.0226397
GOTERM_BP_ALL	GO:0001775~cell activation	4	0.0229807
GOTERM_CC_ALL	GO:0005730~nucleolus	4	0.0253255
GOTERM_CC_ALL	GO:0070013~intracellular organelle lumen	7	0.0281463
GOTERM_BP_ALL	GO:0010557~positive regulation of macromolecule biosynthetic process	5	0.0492617

*Enriched among 41 *PITX2* genes identified by ≥1 array data analysis protocols.

### Oxidative stress and Wnt signaling

**Figure 4:**
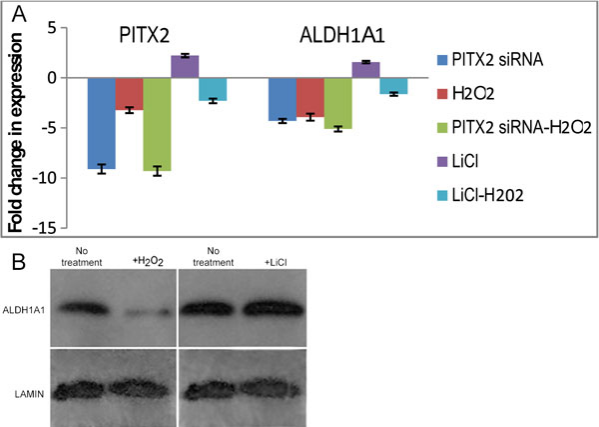
Effects of H_2_O_2_ and LiCl on *ALDH1A1* expression in TM1 cells. **A**: As assessed by real time PCR. See text for details of treatments. Results shown are average of three independent experiments. Two duplicate real time PCRs were performed in each experiment.  Standard deviations are shown.  *β2M* was used as control gene. **B**: As assessed by western blotting.

*PITX2* siRNA decreased *PITX2* (−9.1×) and *ALDH1A1* (−4.3×) expression as already described above (Figure 4A). As *ALDH1A1* is known to be involved in the oxidative stress response of the eye, effect of H_2_O_2_ on *PITX2* and *ALDH1A1* expression was tested. Under the condition of acute exposure to H_2_O_2_ used here, real time PCR showed that it decreased both *PITX2* (−3.2×) and *ALDH1A1* (−3.9×) expression (Figure 4A). Effect of H_2_O_2_ on *ALDH1A1* expression at the protein level was also evident (Figure 4B). Extent of down-regulation of *ALDH1A1* upon simultaneous exposure to *PITX2* siRNA and H_2_O_2_ (−5.1×) was comparable to effect of H_2_O_2_ alone (p=0.566), suggesting that H_2_O_2_ and *PITX2* may affect *ALDH1A1* expression by a common pathway (Figure 4A). It is known that Wnt signaling increases *PITX2* expression in various organs including the eye [45]. Lithium mimics the Wnt pathway by suppressing GSK-3 activity and LiCl is commonly used in experimental settings to produce this effect [38]. Human TM cells possess a functional Wnt signaling pathway and Wnt signaling is implicated in glaucoma [46]. Here, it was observed that LiCl indeed enhanced *PITX2* expression (+2.2×). Furthermore, it seemed to moderately affect increased expression of *ALDH1A1* as well, both at RNA (+1.6×; Figure 4A) and protein (Figure 4B) levels. Pretreatment with LiCl partly prevented H_2_O_2_ induced down-regulation of both *PITX2* (−2.3× versus −3.2×; p=0.226) and *ALDH1A1* (−1.6× versus −3.9×; p=0.076; Figure 4A). Although the differences did not achieve statistical significance, these preliminary results are suggestive and deserve to be pursued. The observations are consistent with the proposal that *ALDH1A1* may at least partly be affected by the Wnt signaling pathway and that effects on *ALDH1A1* expression may be via *PITX2*.

## Discussion

Analysis of microarray gene expression data can be confounded by compound effects of technical parameters causing false positive and false negative identifications and by low but biologically significant differences in expression levels of some genes [47]. With these considerations in mind, we analyzed the array data using three different protocols, the commonly used protocol available in the Limma package and two additional ones that we designed [15]. The likelihood of a selected gene being a true positive was considered to correlate with the number of protocols by which it was selected.  By considering all genes that were selected by at least one protocol, the likelihood of false negatives was expected to decrease. The Limma protocol is based on statistical parameters [15]. Protocols 2 and 3 are both based on fold change in expression levels, with the consideration that genes that show larger fold change are likely to be true positives [47]. Furthermore, data emanating from protocol 1 do not distinguish between comparisons between treated sample and each of two controls separately [15]. Although results on our control repeats showed good reproducibility (only one gene with difference in fold expression >2), even small differences in controls will affect identification of genes affected by siRNA treatments at low (e.g., 2 fold) but possibly biologically significant levels. Protocol 2 produces separate data that reflect effect of *PITX2* siRNA treatments as compared to each of controls. Protocol 3 aims to take into account variations in results between the two different PITX2 siRNAs and the two different TMs. This protocol makes comparisons between *PITX2* siRNA treated cells and average of controls. Although protocols 2 and 3 both consider fold change in expression, they are different as evidenced by incomplete overlap between selected genes. Only 8 of 15 genes selected by protocol 2 are among the 20 genes selected by protocol 3. The Limma protocol selected a larger number of genes than either protocol 2 or 3. We feel most confident that the 5 genes whose expressions were assessed to be affected by *PITX2* knockdown using all three protocols are true positives. Consistent with expectations of our analysis protocol, three of the (*DIRASS3, CXCL6*, and *SAMD5*) five common genes are among the five genes that showed highest fold change in all three protocols (Table 3). Genes that exhibited a lower fold change as result of *PITX2* knockdown were more likely to be selected by only one or two of the protocols. It is expected that at least some genes identified by only one or two of the protocols will prove to be biologically significant.  *ADAMTS5* only identified by protocol 2 and *NOMO2* identified only by protocol 3 may be good candidate genes [17,21,22,48]. *CFL2* which showed 3.2 fold upregulation was not selected by protocol 1. Results of microarray data analysis on several genes, including assessment of absence of effect of knockdown on *ACTB, β2M,* and *GAPDH* by all of the protocols, were confirmed by real time PCR analysis. However, *DKK1* and *KCNJ2* not selected by any of the three protocols were shown to be affected as assessed by real time PCR (Figure 2). This observation is consistent with the notion that there were false negatives. Our experiments do not allow definitive assessment of false positives; however, it was reassuring that results on several TM1 and TM2 microarray based selected genes were confirmed by real time PCR in the two independent TM4 and TM5 cultures (Figure 2). Furthermore, the results on TM4 and TM5 suggest that at least some observed PITX2 effects persist from the second to sixth decade of life. Finally, the observation that a notable number of genes identified here were previously implicated in studies that have biologic relevance to this study is unlikely to be coincidental (Table 5). Our findings for the first time suggest that PITX2 affect the expression of some of these genes.

Two sets of PITX2 related terms identified by the bioinformatics approach are notable, phagocytosis and immune system related functions. Phagocytosis is recognized as an important function of human TM cells, partly because it results in clearing of substances that may hinder facile outflow of aqueous humor [49]. Immune related functions constitute eight of the 29 terms for enriched *PITX2* related functions listed in Table 4, and clinical and experimental studies suggest that immune system functions are involved in glaucoma [50]. Furthermore, immune related functions may be relevant to phagocytosis in the TM [51]. Two genes, *CXCL6* and *ALDH1A1*, were prioritized further analysis.

*CXCL6* codes a pro-inflammatory cytokine that induces directed migration of monocytes and neutrophils [52]. It has been identified to be modulated in three previous glaucoma related global gene analyses [18-20]. Most recently, it was reported that human TM cells secrete significant quantities of CXCL6 [10]. The authors implicated cytokines in regulation of aqueous humor outflow, a function clearly relevant to the glaucoma phenotype [53,54]. Here for the first time we present evidence that PITX2 directly or indirectly affects the expression of this gene. The earlier observations, our finding that *CXCL6* expression is affected by PITX2, and that *PITX2* mutations can cause glaucoma associated ARS suggest that *PITX2* may affect the ARS and glaucoma phenotypes via an effect on the immune response mediated by *CXCL6*.

Down-regulation of *ALDH1A1* and its expression in the human eye, most highly in the corneal epithelium, were demonstrated (Figure 3). ALDH1A1 and ALDH3A1, also expressed in the anterior segment of mammalian eyes, are aldehyde dehydrogenases that minimize the deleterious effects of oxidative damage caused largely by ultraviolet radiation [55]. Highly reactive electrophilic products of lipid peroxidation, 4-hydroxy-2-nonenal (4-HNE) and malon-dialdehyde (MDA), are among the agents that promote oxidative damage [56]. Whereas ALDH1A1 metabolizes both 4-HNE and MDA, MDA is a poor substrate for the more abundant ALDH3A1 [57]. Notably, mouse knockouts of *ALDH1A1* exhibited lens opacity later than *ALDH3A1* and *ALDH1A1* double knockouts [11]. Similarly, some phenotypic consequences in *ALDH1A1* knockouts became evident only at an embryonic stage when *ALDH3A1*was no longer expressed [58]. Late onset glaucoma in some ARS patients harboring *PITX2* mutations may be due to existence of proteins that complement ALDH1A1 activity.

Oxidative stress is an important component in the etiology of glaucoma [59]. Moreover, existence of oxidative stress protection mechanisms and physiologic consequences of oxidative stress in the human TM cells have been reported [60,61]. Treatment of TM cells with a relatively high concentration of H_2_O_2_  caused decreased expression of *PITX2* and *ALDH1A1*, suggesting that *ALDH1A1* is a component of the oxidative response in TM cells and that oxidative stress effects on *ALDH1A1* may be mediated by *PITX2* (Figure 4). Although the data are preliminary, they are consistent with the proposal that down-regulation of *PITX2* causes decreased *ALDH1A1* expression, and this may contribute to evolvement of the ARS phenotype, including its glaucoma related features, via oxidative stress related pathways.

Some of the other genes affected by *PITX2* are relevant to maintenance of homeostasis in TM cells. Expression of *DIRAS3* which is a Rho GTPase exhibited maximum down-regulation by *PITX2* knockdown (Table 3). It has been reported that ectopically expressed PITX2 in HeLa cells profoundly affected the cells’ morphology, migration, and proliferation, and that these effects were mediated through Rho GTPase signaling [62]. Furthermore, myocilin induced loss of actin stress fibers, focal adhesion, and cell matrix cohesiveness in the TM was mediated by Rho GTPase inhibition [63]. *MEIS2*, regulated by *Pax6* and miRNA-204, has roles in vertebrate eye development [64,65]. We show here that *MEIS2* expression is also affected by *PITX2*, suggesting a complicated interaction between PITX2 and PAX6 in directing ocular development. *PITX2* and *MEIS2* were shown to have identical spatial and temporal expression patterns in chicken embryonic facial prominences; cranio-facial anomalies are among the manifestations observed in ARS patients [66].

In conclusion, the results presented suggest that the analysis of the microarray data has led to identification of genes whose expressions are truly affected by PITX2. We expect that the approach used allowed identification of important functions not easily identified by genetic approaches because of their subtle effects and because of existence of compensatory mechanisms. The same approach can be applied for *FOXC1*, which is also mutated in some ARS patients [9]. Addressing the functions and expression patterns of identified genes may lead to better understanding of biochemical and physiologic pathways leading to glaucoma.
